# Functional NK Cell Activation by Ovalbumin Immunization with a Monophosphoryl Lipid A and Poly I:C Combination Adjuvant Promoted Dendritic Cell Maturation

**DOI:** 10.3390/vaccines9101061

**Published:** 2021-09-23

**Authors:** Chau Thuy Tien Le, So Yeon Ahn, Sang-Moo Kang, Eun-Ju Ko

**Affiliations:** 1 Interdisciplinary Graduate Program in Advanced Convergence Technology & Science, Jeju National University, Jeju 63243, Korea; thuytienle102@gmail.com; 2 Department of Veterinary Medicine, College of Veterinary Medicine, Jeju National University, Jeju 63243, Korea; asy0078@gmail.com; 3 Center for Inflammation, Immunity & Infection, Institute for Biomedical Sciences, Georgia State University, Atlanta, GA 30303, USA; skang24@gsu.edu

**Keywords:** natural killer cell, dendritic cell, monophosphoryl lipid A, Poly I:C, adjuvant

## Abstract

Natural killer (NK) cells are one of the types of innate immune cells to remove pathogen-infected cells and modulate inflammatory immune responses. Recent studies have revealed that NK cells could enhance vaccine efficacy by coordinating the innate and adaptive immune responses. In this study, we have evaluated the efficacy of intranasal ovalbumin (OVA) immunization with a monophosphoryl lipid A (MPL) and polyriboinosinic polyribocytidylic acid (poly I:C) combination adjuvant in promoting NK cell recruitment, differentiation, and activation. The frequencies of NK cells were positively correlated with those of dendritic cells (DCs) at the site of immunization. Moreover, the activated NK cells and DCs by the MPL + poly I:C combination adjuvant induced activations of each other cells in vitro. Taken together, this study suggested that the MPL and poly I:C combination adjuvant in OVA vaccination mediated NK cell activation and cellular crosstalk between NK cells and DCs, suggesting a promising vaccine adjuvant candidate for promoting cellular immune responses.

## 1. Introduction

The innate immune system serves as the first line of defense against non-self-antigens invading the body in mammals [1]. Natural killer (NK) cells play a vital role in the innate immune response for the elimination of pathogen-infected cells via secretory and death receptor-mediated pathways and by secreting immunoregulatory cytokines, such as interferon (IFN)-γ and granzyme B [2]. In addition, NK cells are described as the major effector cells toward cancer in innate immunity with a capacity to kill abnormal cells based on levels of major histocompatibility complex-I (MHC-I) expressing on those cells, and whose abilities resemble CD8 T cell functions in adaptive immunity [3,4,5]. Besides their cytotoxic roles, activated NK cells can induce the maturation of dendritic cells (DCs), which drives the development of T helper type 1 (Th1) lymphocytes [6,7]. This interaction between DCs and NK cells plays crucial roles in the innate immune response and the modulation of initial adaptive immune responses. Mature dendritic cells (mDCs) augment the cytotoxicity, production of cytokines, and proliferation of NK cells by producing interleukin (IL)-12 and IL-18 [8,9,10]. NK cells can also undergo differentiation into memory-like NK cells after exposure to these cytokines [11,12]. Moreover, NK cells strongly mediate the maturation of DCs and promote the optimal production of cytokines, such as IL-12 and IL-18, by DCs, which play important roles in the polarization of T cells [10,13]. Additionally, NK cell responses against pathogens decrease the susceptibility of adolescent humans to viral infections [14] as well as the mortality of mice with influenza infection [15]. Collectively, these findings suggest that NK cells not only present as targeting cells in cancer immunotherapy, but also play an important role in determining the efficacy of vaccines.

Vaccines are considered the most effective prophylactic strategies for the prevention of infectious diseases. Typically, vaccines are classified into different types, such as live-attenuated, inactivated, and subunit vaccines. Live-attenuated vaccines can induce strong antigen-specific immune responses; however, they can also cause manifestation of the disease. In contrast, the inactivated whole pathogen vaccines or subunit vaccines are considered relatively safe for children, the elderly, and immunocompromised individuals; however, they elicit weak immune responses [16]. Therefore, adjuvants are often included in vaccine regimens to enhance the immunostimulatory activities and innate immune responses to pathogens, without any side effects or allergic reactions. Aluminum hydroxide (alum)-based compounds, oil-in-water emulsions, and liposome-based adjuvants have been licensed for human use in the past few decades [16,17]. Still, development of novel vaccine adjuvants with better efficacy but without safety concerns are highly demanded.

Innate immune cells express pattern-recognition receptors (PRRs) that recognize a wide variety of infectious agents to recognize any invasion by pathogens. Toll-like receptor (TLR) is one of the most extensively studied PRRs, triggering the innate immune responses and modulating the adaptive immune responses via a variety of cell signaling pathways, such as the myeloid differentiation factor 88 (MyD88) and Toll-IL-1R domain-containing adaptor-inducing IFN-β factor (TRIF)-dependent pathways [18,19]. TLR agonists could be used as potent vaccine adjuvants that can initiate antigen-specific immune responses, stimulate the release of various immunoregulatory cytokines and chemokines, produce high levels of antibodies, and induce apoptosis and phagocytosis [20,21].

Synthetic double-stranded RNA polyriboinosinic polyribocytidylic acid (poly I:C), a TLR3 agonist, induces the activation of the nuclear factor-kappa B (NF-κB) and mitogen-activated protein kinase (MAPK) signaling pathways, produces cytokines via MyD88-dependent or independent signaling pathways, and promotes the maturation of DCs [22]. In a murine model, intranasal co-administration of poly I:C with inactivated influenza hemagglutinin (HA) vaccine induces a higher anti-HA response in the nasal wash and enhances the serum IgG antibody response, thereby providing better protection against an influenza virus infection [23,24]. MPL, a novel licensed adjuvant targeting TLR4, has demonstrated its safety and efficacy in the co-administration with a respiratory syncytial virus (RSV) vaccine at a high dose [25]. A combination of oligodeoxynucleotide CpG and MPL adjuvants, either in respiratory syncytial virus F protein or influenza vaccine, elicited strong antigen-specific immune responses and provided protection against either inflammatory RSV disease or influenza virus infection after a single dose vaccination, further promoting cross-protection against heterosubtypic influenza virus infection [26,27,28].

In this study, we investigated the NK cell responses stimulated by MPL, poly I:C, and combined MPL and poly I:C adjuvants with ovalbumin (OVA) immunization in murine models to validate the cellular mechanisms of the combination adjuvant. Herein, we found that a combination adjuvant of MPL and poly I:C promoted NK cell recruitment to the site of immunization, activation, and immunomodulatory cytokine production compared with OVA only or OVA with single adjuvant groups. Additionally, the DC recruitment at the site of immunization was positively correlated with the NK cell frequencies. Finally, we used an in vitro DC-NK cell co-culture system and found that the DC and NK cells which were stimulated by the combination adjuvant could effectively induce activation of cross each other cells. Collectively, this study provided valuable information that the efficacy of MPL and poly I:C combination adjuvant in OVA vaccination might have been mediated by NK cell activation and cellular crosstalk between NK cells and DCs.

## 2. Material and Methods

### 2.1. Mice and Reagents

Female C57BL/6 (*n* = 6 each group) mice (OrientBio Co., Gyeonggi, Korea) were used in this study. The mice were 6–8 weeks old at the time of priming immunization. OVA was purchased from Sigma-Aldrich (St. Louis, MO, USA), and poly I:C was purchased from InvivoGen (San Diego, CA, USA). All reagents were prepared according to the manufacturer’s instructions.

### 2.2. Mice Immunization

Before the intranasal administration of OVA and adjuvants, the female C57BL/6 mice were anesthetized with isoflurane (Hana Pharm Co., Gyeonggi, Korea) using an oxygen controlling machine and then intranasally administered: 50 μL of PBS containing 10 μg of OVA, 50 μL containing 10 μg of OVA + 1 μg of MPL, 50 μL of PBS containing 10 μg of OVA + 10 μg of poly I:C, or 50 μL of PBS containing 10 μg of OVA + 1 μg of MPL + 10 μg of poly I:C at day 0 (prime) and day 14 (boost).

### 2.3. Sample Harvest and Preparation

The mice in each group were euthanized at the indicated dates post-immunization (prime D1, boost D1, and boost D14). The bronchoalveolar lavage fluid (BALF) was collected from the trachea using an 18-gauge Excel Safelet Catheter and phosphate-buffered saline (PBS). The BALF was then centrifuged at 1600 rpm at 4 °C for 5 min. The supernatant was stored at −20 °C for cytokine enzyme-linked immunosorbent assay (cytokine ELISA), and the cell pellet was resuspended in 2% fetal bovine serum (FBS) containing PBS (fluorescence-activated cell sorting (FACS) buffer) for the analysis of the cell phenotype. The lungs and spleens were harvested aseptically from immunized-mice and homogenized in the Roswell Park Memorial Institute (RPMI) medium 1640 (Fisher Scientific, Corning, NY, USA) using a 70 μm cell strainer. The homogenates were then centrifuged at 1600 rpm at 4 °C for 5 min, and the supernatant was stored at −20 °C for cytokine ELISA. Next, the red blood cells (RBCs) were removed from the cell pellets, and the single cells were used for FACS and other assays.

### 2.4. Flow Cytometry

The prepared lung and BAL cells were stained with different surface markers for NK cells, including Live/Dead-Amcyan (LIVE/DEAD™ Fixable Aqua Dead Cell Stain Kit; Thermo Fisher Scientific, Waltham, MA, USA), CD3-BV421 (clone 17A2; BD Horizon™, Piscataway, NJ, USA), CD45-PerCP (clone 30-F11; BD Pharmingen™, Franklin Lakes, NJ, USA), CD49b-PE (clone HMα2; BD Pharmingen™), CD107a-APC (clone 1D4B; BD Pharmingen™, Franklin Lakes, NJ, USA), CD69-AF700 (clone H1.2F3; BD Pharmingen™); CD11b-APC/Cy7 (clone M1/70; BD Pharmingen™, Franklin Lakes, NJ, USA), and CD27-PE/Cy7 (clone LG.3A10; BD Pharmingen™, NJ, USA). For intracellular cytokine staining, the cells were incubated with BD GolgiStop for 5 h, stained with the surface marker antibodies, and then fixed and permeabilized using a Fixation/Permeabilization Solution Kit (BD Biosciences). IFN-γ-APC/Cy7 (clone XMG1.2, BD Pharmingen™, Franklin Lakes, NJ, USA) was used for intracellular staining. Flow cytometry data were acquired using a BD LSR flow cytometer and analyzed using the FlowJo Software Program (Tree Star Inc., Ashland, OR, USA). The gating strategy is shown in Supplementary Figure S1.

### 2.5. Cytokine ELISA

The levels of IFN-γ (R&D Systems, Minneapolis, MN, USA), granzyme B (R&D Systems, Minneapolis, MN, USA), IL-12p70 (R&D Systems, Minneapolis, Minnesota, USA), IL-12p40 (Invitrogen, Waltham, MA, USA), IL-15 (Invitrogen, Waltham, MA, USA), and IL-18 (R&D Systems, Minneapolis, MN, USA) in the BALF, lung extract, and cell supernatants were measured using ELISA kits, following the manufacturer’s instructions.

### 2.6. Preparation of Resting and Activated NK Cells

NK cells were isolated from the spleens of C57BL/6 mice via negative magnetic selection using the EasySep™ mouse NK Cell Isolation Kits and EasySep™ Magnet (STEMCELL Technologies Inc., Vancouver, BC, Canada). CD3^−^CD49b^+^ NK cell purity was 94.1% after the NK cell isolation from the spleen cells, but CD3^+^ T cells were reduced from 27.3% to 0.28% after the isolation (Supplementary Figure S2). To generate the activated NK (ANK) cells, purified NK cells (5 × 10^5^ cells/mL) were cultured in 6-well plates for 48 h in 10% FBS, 1× antibiotics, 1× sodium pyruvate, 1× non-essential amino acids, and 1 × 2-mercaptoethanol containing RPMI 1640 media with GlutaMax (10% LCM) in the presence of 100 ng/mL MPL, 1000 ng/mL poly I:C, the combination of 100 ng/mL MPL and 1000 ng/mL poly I:C, and only the medium. The ANK cells were then harvested, washed to remove all excess reagents, and resuspended in 10% LCM before use in future experiments.

### 2.7. Preparation of Immature and Mature DCs

Bone marrow cells were collected from the femur and tibia of C57BL/6 mice and the RBCs were removed. Then, the bone marrow cells were cultured in the presence of 20 ng/mL of recombinant mouse granulocyte-macrophage colony-stimulating factor (mGM-CSF) at 37 °C with 5% carbon dioxide (CO_2_). The culture medium containing mGM-CSF was replaced every 2 d. After 6 d of culture, the immature DCs were harvested by pipetting and seeded at a concentration of 5 × 10^5^ cells/mL in 6-well plates. Thereafter, 100 ng/mL MPL, 1000 ng/mL poly I:C, the combination of 100 ng/mL MPL and 1000 ng/mL poly I:C, and only the medium were treated and cultured for 2 d to facilitate the maturation of DCs.

### 2.8. In Vitro NK Cell-Mediated Maturation of DCs and Production of IL-12p70 and IL-18

Immature DCs (iDCs, 2 × 10^5^ cells/mL) and ANK cells (4 × 10^5^ cells/mL) were co-cultured at a ratio of 1:2 in the 96-well U-bottomed plate. After 2 d of incubation, the cultured cells were harvested. The supernatant was stored for cytokine ELISA and the cells were resuspended in FACS buffer and stained with different surface markers, including Live/Dead-Amcyan (Thermo Fisher Scientific), CD49b-PE (BD Pharmingen^TM^), CD69-AF700 (BD Pharmingen^TM^, Franklin Lakes, NJ, USA), CD11c-PE/Cy7 (Monoclonal Antibody N418; Thermo Fisher Scientific, Waltham, MA, USA), CD40-BV605 (clone 3/23; BD Biosciences, Franklin Lakes, NJ, USA), and CD86-FITC (clone GL1; BD Pharmingen^TM^). Flow cytometry data were acquired using a Becton-Dickenson LSR flow cytometer and analyzed using the Flowjo Software Program (Tree Star Inc., Ashland, OR, USA).

### 2.9. In Vitro mDC-Mediated Activation of NK Cells and Production of IFN-γ and Granzyme B

Resting NK cells (2 × 10^5^ cells/mL) and mDCs (1 × 10^6^ cells/mL) were co-cultured at a ratio of 1:5 in the 96-well U-bottomed plate for 2 d. The cultured cells were then harvested, and the supernatant was stored for cytokine ELISA. Then, the cells were resuspended in FACS buffer, which was stained with different surface markers, including Live/Dead-Amcyan (Thermo Fisher Scientific), CD49b-PE (BD Pharmingen^TM^), CD69-AF700 (activation marker for NK cells; BD Pharmingen^TM^), CD11c-PE/Cy7 (Thermo Fisher Scientific), CD40-BV605 (Thermo Fisher Scientific), and intracellular cytokine marker IFN-γ-APC/Cy7 (BD Pharmingen^TM^). The cell phenotypes and the frequency of IFN-γ were determined using the Becton-Dickenson LSR flow cytometer and analyzed using the Flowjo Software Program (Tree Star Inc., OR, USA).

### 2.10. Statistical Analysis

All data were statistically analyzed using the GraphPad Prism 9^®^ software (GraphPad Software, Inc., San Diego, CA, USA).

## 3. Results

### 3.1. NK Cells Were Recruited to the Site of Immunization after the Prime and Boost Dose Inoculation of OVA with the MPL and Poly I:C Adjuvants

**Figure 1:**
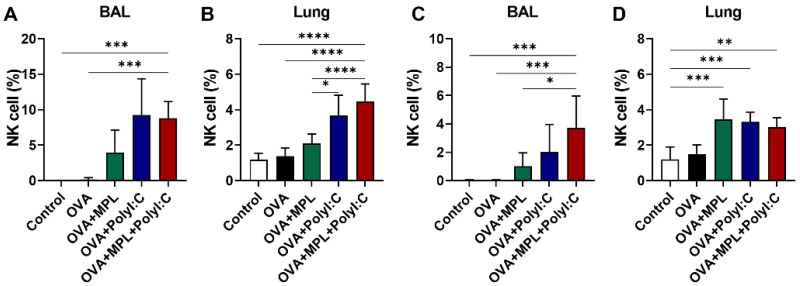
NK cell recruitment after intranasal immunizations of OVA with adjuvants. C57BL/6 mice were intranasally immunized with ovalbumin alone or plus MPL, poly I:C or MPL + poly I:C. The immunizations were given two times with 2 weeks interval (prime and boost). At day 1 post prime (**A**,**B**) and boost (**C**,**D**) immunizations, NK cell population in BAL and Lung cells were analyzed by flow cytometry. CD45^+^CD3^−^CD49^+^ cells were gated as NK cells. All data show the mean ± SD. Statistical analysis between groups were performed by One-way ANOVA and Tukey’s multiple comparison test. * *p* < 0.0332, ** *p* < 0.0021, *** *p* < 0.0002 and **** *p* < 0.0001.

NK cells play vital roles in innate immune responses owing to their capacity for lysis and for modulating the adaptive immunity via the release of immunoregulatory cytokines [29]. Here, we observed the recruitment of NK cells in BAL and lung samples one day after prime and boost intranasal OVA with adjuvant inoculations (Figure 1). During the immunizations, any side effects such as body weight loss and respiratory symptoms were not observed. Poly I:C and the combination of MPL + poly I:C-adjuvanted OVA immunization significantly enhanced the NK cell frequencies in both BAL and lung samples compared with the other groups. There was no additive or synergic effect of MPL and poly I:C combination on NK cell recruitment compared to poly I:C adjuvanted group. The NK cell frequencies in BAL and lung samples showed a decreased trend after the boost immunization compared to those of the prime immunization, but there was no significant difference. The immunization with OVA only did not induce NK cell recruitment at the site of immunization, while the OVA + MPL immunization promoted NK cell recruitment to the lungs on day one post-boost immunization (Figure 1D). These data suggest that poly I:C effectively enhanced NK cell recruitment at the site of immunization.

### 3.2. Combination of MPL + Poly I:C Adjuvant OVA Inoculation Enhanced the Maturation and Activation of NK Cells

**Figure 2:**
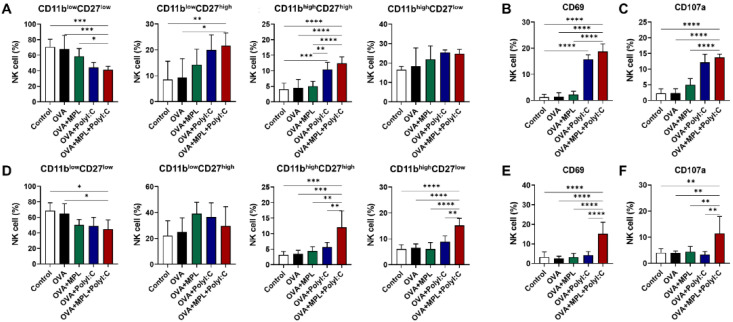
NK cell differentiation and activation marker expressions in lung after intranasal immunizations of OVA with adjuvants. Lung samples were harvested at day 1 post prime and boost vaccinations. (**A**–**C**) NK cell subsets and activation marker expressions after the prime immunization. (**D**–**F**) NK cell subsets and activation marker expressions after the boost immunization. The results show the mean ± SD. Statistical analysis between groups were performed by One-way ANOVA and Tukey’s multiple comparison test. * *p* < 0.0332, ** *p* < 0.0021, *** *p* < 0.0002 and **** *p* < 0.0001.

Murine NK cells are further differentiated based on the expression levels of CD11b and CD27. The CD11b^low^CD27^low^ NK population is considered an immature NK cell phenotype, while the CD11b^low^CD27^high^ NK cells are known to have a high potential for proliferation. CD11b^high^CD27^high^ and CD11b^high^CD27^low^ NK cells are the most mature NK cells, with limited proliferation capabilities but increased cytokine production and strong effector functions [30]. We found that the level of CD11b^low^CD27^low^ NK cell subset was decreased, while the levels of CD11b^low^CD27^high^, CD11b^high^CD27^high^, and CD11b^high^CD27^low^ NK subsets were increased in all the adjuvanted groups after both prime and boost immunizations (Figure 2A,D). In particular, after the prime immunization, the poly I:C and MPL + poly I:C combination adjuvant induced CD11b^high^CD27^high^ NK cells significantly compared with that in the Control, OVA-only or MPL-adjuvanted OVA groups (Figure 2A). After boost immunization, however, MPL + poly I:C combination adjuvant elicited significant NK cell maturation, not only CD11b^high^CD27^high^ but also CD11b^high^CD27^low^ (Figure 2D).

Additionally, we analyzed the expression levels of the activation markers on the total NK cells from lungs after prime (Figure 2B,C) and boost (Figure 2E,F) immunizations. CD69 is a general NK cell activation marker and CD107a is a degranulation marker, which are increased when NK cells become functionally activated [31]. OVA-only or OVA + MPL immunization did not affect the expression levels of the activation markers on NK cells. Poly I:C-adjuvanted OVA immunization enhanced the expression levels of both CD69 and CD107a after the prime immunization, but not after boost immunization. NK cells from the MPL + poly I:C combination adjuvant group exhibited significantly enhanced expression levels of CD69 and CD107a after the boost as well as the prime immunization. The matured NK cell subsets (CD11b^high^CD27^high^ and CD11b^high^CD27^low^ NK cells) also exhibited a similar trend of activation marker expression as the total NK cells (Supplementary Figure S3). After prime immunization, poly I:C and MPL + poly I:C adjuvants enhanced CD69 and CD107a expressions on both CD11b^high^CD27^high^ and CD11b^high^CD27^low^ NK cells, but after boost immunization, only MPL + poly I:C could elicit the NK cell activation marker expressions. These data indicate that the MPL + poly I:C combination adjuvant significantly enhances the stimulation and maturation of NK cells at the site of immunization.

### 3.3. MPL + Poly I:C Adjuvanted OVA Immunization Promoted the Production of Cytokines by NK Cells in the Lungs

**Figure 3:**
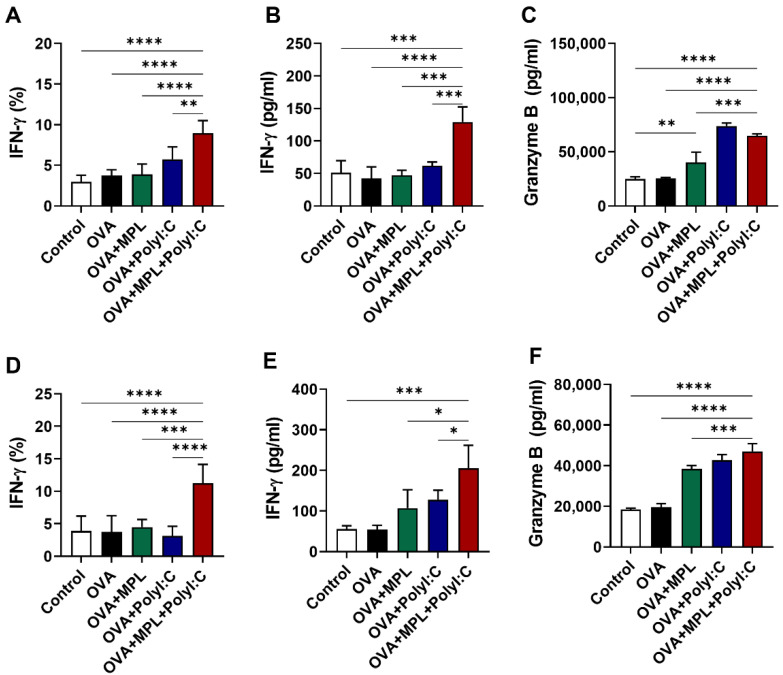
Cytokine production after intranasal immunizations of OVA with adjuvants. Lung samples were collected after one day post prime (**A**–**C**) and boost (**D**–**F**) immunizations. (**A**,**D**) The frequencies of IFN-γ-positive NK cells were analyzed by flow cytometry. (**B**,**C**,**E**,**F**) The concentration of IFN-γ and Granzyme B in lung homogenates were measured by ELISA. The data show the mean ± SD. Statistical analysis between groups were performed by one-way ANOVA and Tukey’s multiple comparison test. * *p* < 0.0332, ** *p* < 0.0021, *** *p* < 0.0002 and **** *p* < 0.0001.

We next examined the effects of adjuvants on the secretion of cytokines by NK cells in the vaccinated mice. IFN-γ-producing NK cells in the lungs were analyzed by intracellular cytokine staining and flow cytometry (Figure 3A,D), while the IFN-γ and granzyme B secretions in the lung homogenates were determined by ELISA (Figure 3B,C,E,F). Similar to the expression levels of the activation markers on NK cells, the poly I:C adjuvant increased IFN-γ-producing NK cells after prime immunization (Figure 3A), but not after boost immunization (Figure 3D). In contrast, the MPL + poly I:C combination adjuvant enhanced IFN-γ-producing NK cell frequencies in both prime and boost immunizations (Figure 3A,D). The production of IFN-γ in the lung homogenates was induced by the MPL + poly I:C adjuvant in both prime and boost immunizations (Figure 3B,E), while the production of granzyme B was promoted by the MPL, poly I:C, and the combined MPL + poly I:C adjuvants (Figure 3C,F). We also performed an NK cell-mediated cytotoxicity assay via the co-culture with YAC-1 cells to elucidate the functional activation of NK cells after OVA immunization with adjuvants, Poly I:C and the combination of MPL + poly I:C groups showed the highest cytotoxic activities both in vitro and in vivo (Supplementary Figure S4). Collectively, we found that the combination of MPL + poly I:C was highly effective in the production of cytokines by immune cells, including NK cells, and also aided in enhancing NK cell activation.

### 3.4. The Frequencies of NK Cells and DCs Were Found to Be Correlated after In Vivo Immunizations

**Figure 4:**
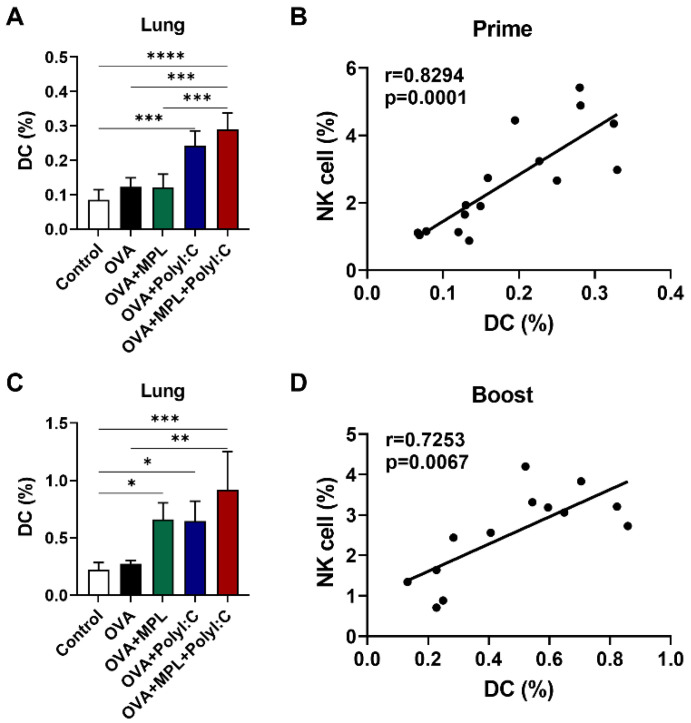
Correlation between DC and NK cell recruitment after intranasal immunizations of OVA with adjuvants. Lung samples of the immunized mice were harvested at day one prime (**A**,**B**) and boost (**C**,**D**) immunizations and then cell phenotypes were identified by flow cytometry. The frequencies of DC were analyzed as CD45^+^F4/80^−^CD11c^+^MHCII^high^ population. The correlation of NK cell (Figure 1B,D) and DC frequencies were analyzed. The data show the mean ± SD. Statistical analysis between groups were performed by One-way ANOVA and Tukey’s multiple comparison test. * *p* < 0.0332, ** *p* < 0.0021, *** *p* < 0.0002 and **** *p* < 0.0001. For correlation analysis, Spearman’s rank correlation coefficients (r) and *p* value are calculated and shown.

NK cells and DCs can interact with each other to regulate their activation by producing cytokines, and this interaction can influence the vaccine and vaccine adjuvant efficacy [9,13]. DCs initiate antigen-specific adaptive immune responses after the uptake and activation of antigens [7,32,33]. To evaluate the correlation between NK cells and DCs after the adjuvanted immunizations, we analyzed the total DC populations in the lungs after vaccination and performed correlation analysis between NK cells and DC populations. We found that the DC populations were significantly increased in the poly I:C and MPL + poly I:C adjuvanted groups after prime immunization (Figure 4A) and boost immunization with MPL, poly I:C, and MPL + poly I:C groups significantly enhanced the DC frequencies in the lung (Figure 4C). Strong correlations between DCs and NK cells were found after both prime and boost immunizations (r = 0.8294 and r = 0.7253, respectively) (Figure 4B,D).

### 3.5. The Combination of MPL + Poly I:C-Treated NK Cells Can Induce the Maturation of DCs and Enhance the Cytokine Production In Vitro

**Figure 5:**
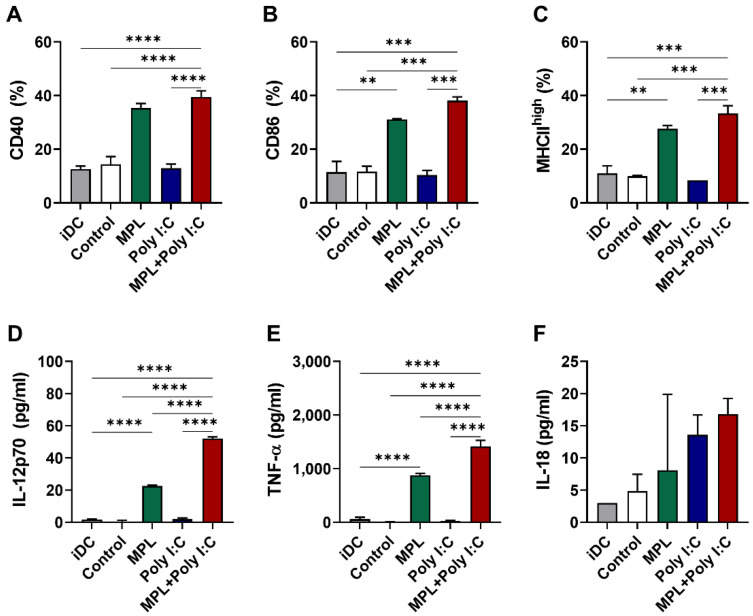
In vitro activation of immature DCs by adjuvant-activated NK cells. Immature DCs were co-cultured with adjuvants pre-treated NK cells isolated from spleen cells of naïve C57BL/6 mice. After two days of co-culture, the cells were harvested and analyzed with the DC activation marker expressions by flow cytometry. The culture supernatants were used for measurement of cytokine production. The percentages of CD40 (**A**), CD86 **(B**), and MHCII^high^ (**C**) expression on DCs co-culture with the activated NK cells. The level of IL-12p70 (**D**), TNF-α (**E**), and IL-18 (**F**) were measured by ELISA. The data show the mean ± SD. Statistical analysis between groups were performed by one-way ANOVA and Tukey’s multiple comparison test. ** *p* < 0.0021, *** *p* < 0.0002 and **** *p* < 0.0001.

To investigate whether the adjuvant-activated NK cells would induce the maturation of DCs and production of cytokines, NK cells were freshly purified from the C57BL/6 mice for in vitro functional analysis. The isolated NK cell purity was 94.1% (Supplementary Figure S2). The NK cells were pre-stimulated with either MPL, poly I:C, or MPL + poly I:C for two days for activation. IFN-γ production was significantly elevated in the MPL, poly I:C, and MPL + poly I:C treated NK cells, and granzyme B was secreted from the MPL, and MPL + poly I:C treated NK cells (Supplementary Figure S5). The activated NK cells were then co-cultured with the immature bone marrow-derived DCs (BMDCs). After two days of co-culture, the expression levels of the DC activation markers and the production of cytokines were determined. The DCs co-cultured with MPL alone and the MPL + poly I:C pretreated NK cells showed increased expression levels of CD40, CD86, and MHC II on their surfaces (Figure 5A–C). The DCs co-cultured with MPL + poly I:C pre-treated NK cells exhibited the highest levels of secretion of IL-12p70, IL-18, and TNF-α (Figure 5D–F). These data suggest that the NK cells activated by MPL + poly I:C pre-treatment can effectively induce the maturation of DCs and the production of cytokines.

### 3.6. The Maturation of DCs by Adjuvant Treatment Can also Trigger the Activation of NK Cells In Vitro

**Figure 6:**
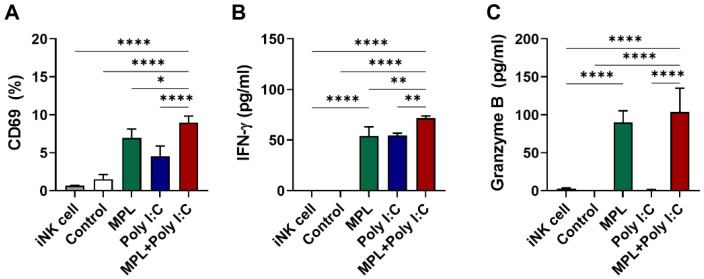
In vitro activation of immature NK cell from naïve mice by adjuvant pre-treated matured DCs. BMDCs were pre-activated by MPL, poly I:C, or MPL + poly I:C for two days, and then co-cultured with NK cell isolated from spleen cells of naïve C57BL/6 mice for two days. CD69 NK activation marker expressions were analyzed by flow cytometry (**A**), and IFN-γ (**B**) and Granzyme B (**C**) levels in culture supernatant were determined by ELISA. The data show the mean ± SD. Statistical analysis between groups were performed by one-way ANOVA and Tukey’s multiple comparison test. * *p* < 0.0332, ** *p* < 0.0021, and **** *p* < 0.0001.

To assess whether the maturation of DCs via pretreatment with adjuvants influenced the activation of NK cells and their production of cytokines, we directly co-cultured the adjuvant-pretreated BMDCs with the splenic NK cells isolated from naïve mice in vitro. NK activation markers and the production of cytokines were also measured (Figure 6). Similar to the ANK-induced maturation of DCs in vitro, the MPL + poly I:C pretreated DCs induced significantly enhanced the CD69 expression on NK cells (Figure 6A) as well as the production of IFN-γ (Figure 6B) and granzyme B (Figure 6C). These data suggest that the combination of MPL + poly I:C is efficient in strengthening NK-DC interaction under both in vitro and in vivo conditions.

## 4. Discussion

Recently, many infectious diseases, including the coronavirus disease 2019 (COVID-19) and influenza, have posed a threat to public health worldwide, and the most effective method to prevent the occurrence of these diseases is vaccination. Despite the continuous research efforts to develop new vaccines, most vaccine and vaccine adjuvant candidates encounter several hurdles, including the genetic mutations of the pathogens, low efficacies of the vaccines, and unexpected side effects [16,21,32,33,34]. Therefore, it is important to develop safe and effective vaccines and vaccine adjuvants to provide protection against these pathogens.

In this study, we tested the hypothesis that the combination of MPL and poly I:C might be a vaccine adjuvant candidate to enhance the immune responses, specifically NK cell responses, even at low doses. We found that the combined adjuvant of MPL (1 μg) and poly I:C (10 μg) promoted the activation, differentiation, and cytokine production of NK cells. It also strengthened the interaction between NK cells and DCs compared with that in the single-adjuvanted OVA or OVA-only immunization groups.

MPL and poly I:C combination adjuvant elicited more NK cell recruitment to the airway and lung one day after intranasal inoculations, and triggered NK cell maturation expressing high levels of CD11b. As demonstrated by previous studies [30,31], the different expression of CD11b and CD27 on NK cells indicated different functional potentials. The CD11b^high^ NK cell subsets showed a greater effector function, chemokine responsiveness, and cytokine production, whereas CD11b^low^ NK cells displayed a high proliferation capacity [30,35,36]. Lung NK cells from the MPL + poly I:C adjuvanted OVA-immunized mice exhibited a significant increase in CD69 and CD107a activation marker expression as well as CD11b expression, cytokine production such as IFN-γ and granzyme B, and cytotoxic functions. Our data indicated that the NK cell recruitment at the site of immunization was enhanced by poly I:C, but the activation, differentiation, and cytokine production of NK cells were significantly elevated by MPL + poly I:C combination, suggesting a positive role of the adjuvant combination. NK cells exhibited different recruitment and activation trends after the prime and boost immunizations. This might be due to existing adaptive immune responses developed after the prime immunization. Pre-existing immune responses such as antibodies and memory T cells could affect the innate immune responses resulting in rapid activation of NK cells after booster dose. Alternatively, it might be due to memory-like NK cell responses. Recent studies have suggested that innate immune cells also have memory-like properties, showing different cytokine production and activation marker expressions upon antigen re-encounter. These memory-like innate cell responses were induced by cytokines and activating NK cell receptor pathways [37]. However, the results in this study do not provide evidence for the memory-like NK cell activation after boost inoculation with MPL + poly I:C combination adjuvant.

NK cells also interact with other immune cells to prime and induce immune responses [13]. The collaboration of NK cells and DCs is crucial for the stimulation of the innate immune responses as well as the modulation of the initial adaptive immunity via the release of immune-regulatory cytokines, including IFN-γ, TNF-α, IL-12, IL-15, and IL-18 [8,12]. IFN-γ-producing NK cells regulate the innate resistance against pathogens by activating the phagocytic cells and priming antigen presenting cells (APCs) to produce IL-12p70, which is a major Th1-driving cytokine [38]. IL-15 produced by APCs affects the proliferation and functional maturation of NK cells as well as the development of memory CD8 T cells [35,36,39]. IL-18 is an IFN-γ-inducing factor and a potent immune-regulatory cytokine for the activation of NK cells and production of cytokines [12,13]. Moreover, the cooperation between IL-12 and IL-18 enhances the activation, proliferation, and cytotoxicity of NK cells [11]. Furthermore, these IL-12/15/18-preactivated NK cells were demonstrated to appear as cytokine-induced memory-like (CIML) NK cells, capable of inducing an enhanced cytokine production and proliferation after restimulation [40,41]. In addition, CIML NK cells were proved to be useful in elimination of malignancies in murine model and are being tested in cancer immunotherapy for leukemia in humans [42,43]. In this study, we observed that both NK cells and DCs were recruited by the MPL + poly I:C adjuvanted OVA immunization and the frequencies of NK cells and DCs were positively correlated in the lungs after the prime and boost immunizations (Figure 4). Moreover, the levels of IL-12p40, IL-15, and IL-18 were significantly elevated in the lung extracts after the MPL + poly I:C adjuvanted OVA immunization (Supplementary Figure S6), resulting in triggering NK cell maturation expressing activation markers and releasing cytokines (Figure 2 and Figure 3), and also inducing memory CD8^+^ T cells producing specific IFN-γ [44]. In addition to these in vivo observations, we confirmed that the activated NK cells and DCs by the adjuvant treatment further elicited the activation of iDCs and NK cells, respectively, in an in vitro co-culture system. As a result, the adjuvanted OVA immunization, especially with the combination adjuvants of MPL + Poly I:C significantly enhanced presentation of Ag to CD4^+^ T cells, leading to strengthening B cells to produce IgG, IgG1 and IgG2c antibodies against OVA and inducing antigen-specific memory T cell responses (a manuscript under review). This underlying cellular mechanism by which MPL and poly I:C enhanced the activation of innate immune cells, including NK cells and DCs, might promote further local and systemic immune responses specific for the antigen.

The safety of a new vaccine adjuvant is a critical factor that determines its potential use in the future. Lowering the adjuvant doses is an easy way to reduce the undesirable side effects; however, this can also affect the efficacy of the adjuvant [25,45]. To overcome this hurdle, many researchers have analyzed the efficacies of various combinations of vaccine adjuvants. A mixture of vaccine adjuvants with different immune-regulating mechanisms could enhance the immune responses by stimulating different immune receptors and signaling pathways [19,21]. AS04, a licensed human vaccine adjuvant, consists of a combination of MPL and alum, which trigger the TLR4 and nucleotide-binding oligomerization domain-like receptor (NLR) family pyrin domain-containing 3 (NLRP3) pathways, respectively [19,46,47]. In this study, we evaluated the efficacy of the combination adjuvant vaccine at low doses, consisting of 1 μg of MPL and 10 μg of poly I: C. We found that this combination exhibited synergistic or additive effects on the activation of innate immune cells and their functions at the site of immunization, without apparent adverse effects. Taken together, our results indicate that MPL and poly I:C at low doses can be used as a potential vaccine combination adjuvant to induce the activation of innate immune cells, including NK cells and further CIML NK cells.

NK cells are known to develop normally in recombination activating genes 1 and 2 (Rag1/2) deficient mice where T cells and B cells are absent as reported in earlier studies [48,49,50]. Nonetheless, a later study reported that RAG-deficient NK cells exhibited a diminished survival capacity upon virus-driven proliferation, as well as defects in repairing DNA breaks and DNA damage response mediators, suggesting a new role of RAG in NK cell biology [51]. Thus, use of Rag1/Rag2 knockout mice to exclude the potential contributions of T cells might compromise the intrinsic functions of NK cells. TLR3 and TLR4 are expressed on multiple immune cells including NK cells and dendritic cells (DCs), mediating activation via TRIF-dependent and MyD88-dependent signaling pathways [18,19]. Further investigating the effects of combination adjuvants in RAG1/2 knockout and TLR knockout mice will supplement the cellular and molecular mechanisms underlying the functions of this combined MPL + poly I:C TLR agonist adjuvant. In addition, the adjuvant effects of MPL + poly I:C are to be tested with clinically relevant practical vaccine antigens (other than OVA) to broaden its application and analyze its efficacy in other animal models.

## Data Availability

The data presented in this study are available on request from the corresponding author.

## Supplementary Materials

The following are available online at https://www.mdpi.com/article/10.3390/vaccines9101061/s1, Figure S1: Gating strategy of flow cytometry, Figure S2: NK cell purity after NK isolation by negative selection, Figure S3: Activation marker expression levels on the mature NK cell populations in lung after intranasal immunizations of OVA with adjuvants, Figure S4: Cytotoxicity mediated by NK cells after in vitro adjuvant treatment and in vivo immunizations with OVA + adjuvants, Figure S5: Cytokine production by isolated NK cells after in vitro adjuvant treatment, Figure S6: Induction of cytokine production after stimulation by MPL and Poly I:C in the lungs of C57BL/6 mice at prime and boost dose.
