# Supplementary material for: Functional NK Cell Activation by Ovalbumin Immunization with a Monophosphoryl Lipid A and Poly I:C Combination Adjuvant Promoted Dendritic Cell Maturation
Source: Vaccines (Basel). 2021 Sep 23;9(10):1061. doi: 10.3390/vaccines9101061 (PMC8540815; doi:10.3390/vaccines9101061)
Supplement: Supplementary file 1 [file vaccines-09-01061-s001.zip › vaccines-1365424-supplementary.pdf]

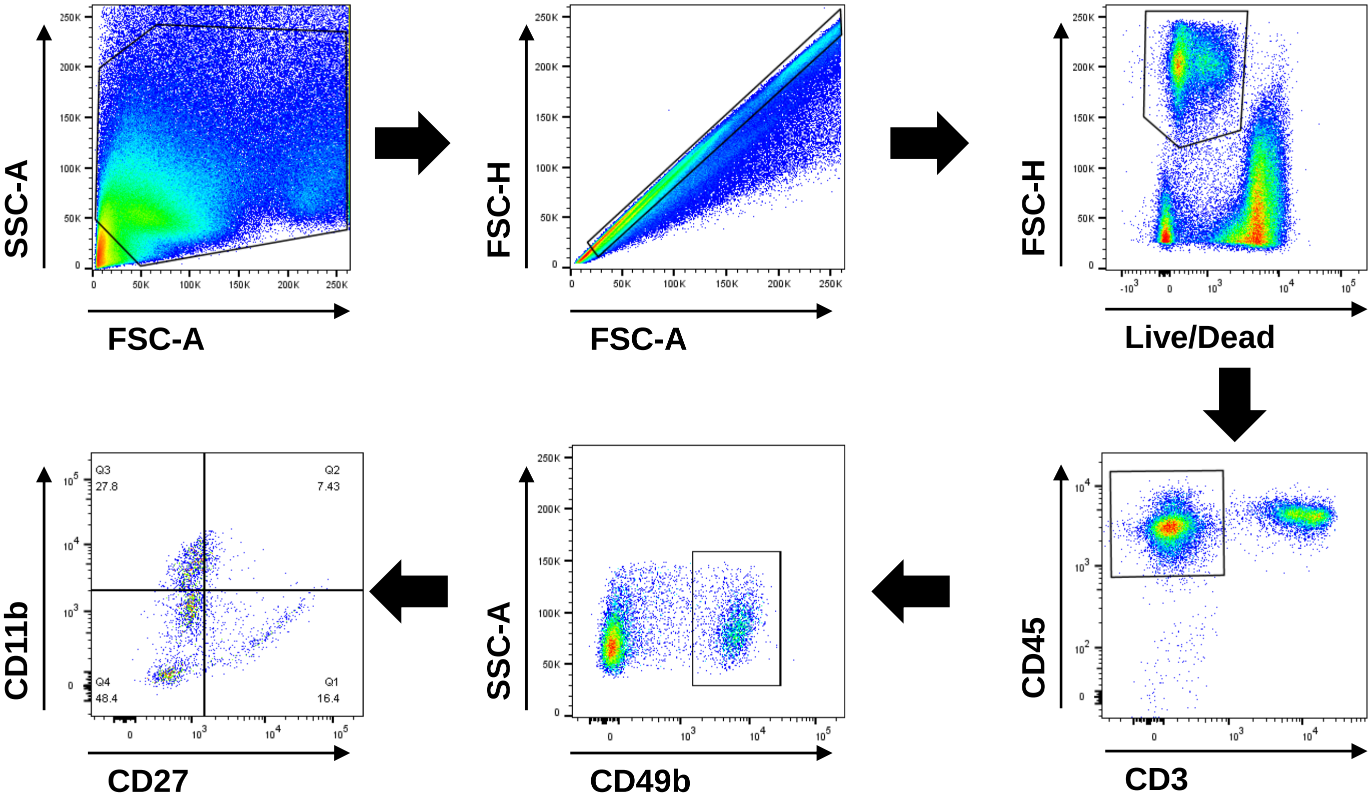

Supplementary Figure S1. Flow cytometry gating strategy. Gating strategy of NK cells ($CD45^+CD3^-CD49b^+$) and NK cell subsets by CD27 and CD11b expressions.

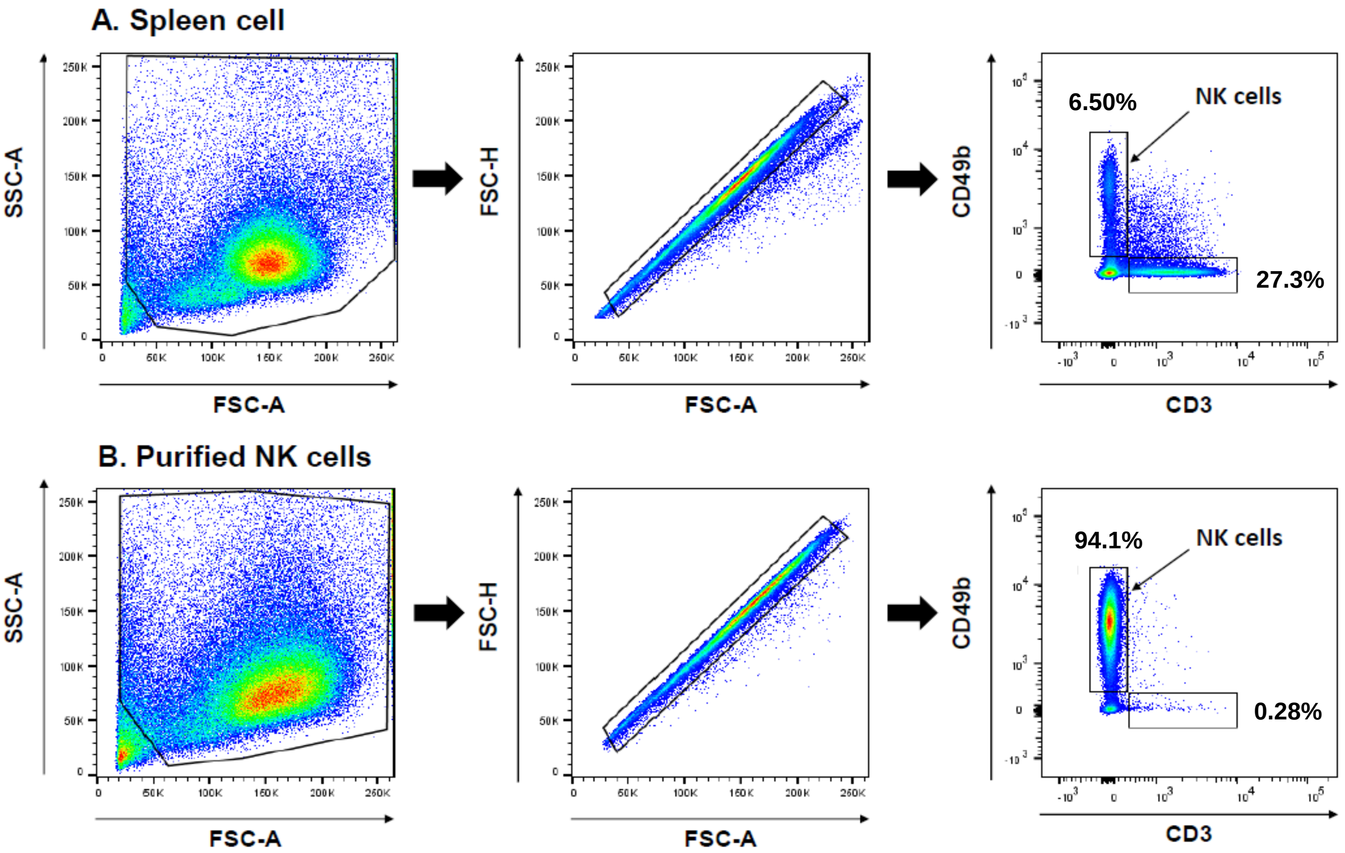

Supplementary Figure S2. NK cell purity after NK isolation by negative selection. NK cells ($CD3^{-}CD49b^{+}$) and T cell ($CD3^{+}CD49b^{-}$) percentages in total spleen cells (**A**) and after NK isolation (**B**) were shown.

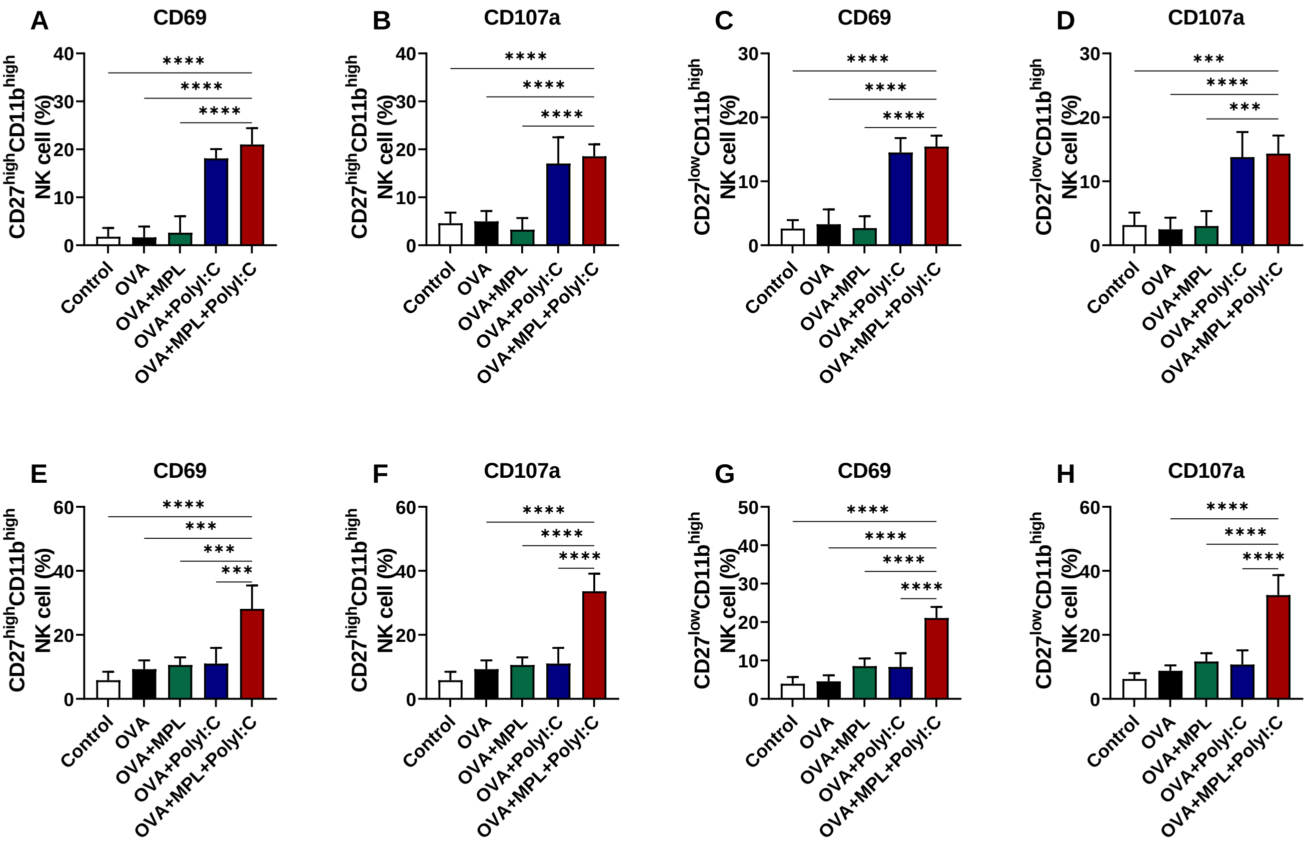

Supplementary Figure S3. Activation marker expression levels on the mature NK cell populations in lung after intranasal immunizations of OVA with adjuvants. Lung samples were harvested at day 1 post prime and boost vaccinations. The matured NK cells were gated as $CD27^{high}CD11b^{high}$ or $CD27^{low}CD11b^{high}$ from $CD45^{+}CD3^{-}CD49b^{+}$ total NK cells. Activation marker expressions by mature NK cells after the prime immunization **(A-D)** , and After the boost immunization **(E-H)** were shown in the mean ± SD. Statistical analysis between groups were performed by One-way ANOVA and Tukey's multiple comparison test. ***; p<0.0002 and ****; p<0.0001.

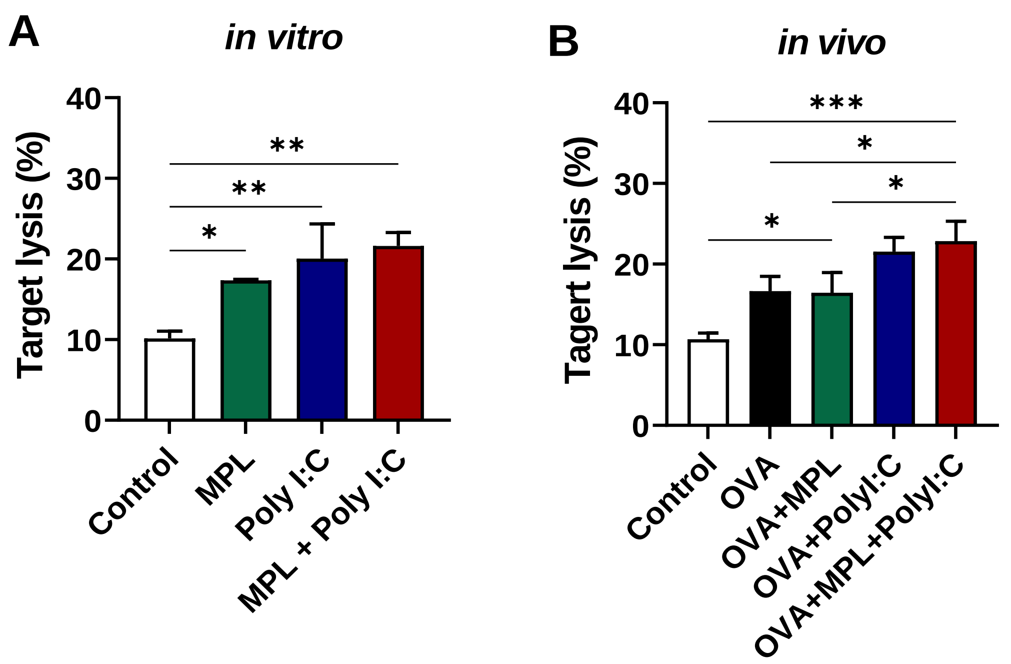

Supplementary Figure S4. Cytotoxicity mediated by NK cells after *in vitro* adjuvant treatment and *in vivo* immunizations with OVA+adjuvants. (A) NK cells were isolated from spleen cells of naïve mice and then treated with MPL, Poly I:C, and MPL+PolyI:C for 2 days. **(B)** NK cells were isolated from spleen cells of naïve or immunized mice at day 1 post boost immunization. The effector cells (E), *in vitro* adjuvant pre-treated-NK cells and the *in vivo* isolated-NK cell from the immunized mice, were stained with 2000 nM carboxyfluorescein succinimidyl ester (CFSE) and then cultured with target cells (T), YAC-1 cells, with a ratio of 5 (E) : 1 (T). Cells were collected after 4-hour incubation and the dead cell frequencies of target cells ($CFSE^{-}7\text{-}AAD^{+}$ YAC-1 cells) were determined by flow cytometry. The data show the mean ± SD. Statistical analysis between groups were performed by One-way ANOVA and Tukey's multiple comparison test. *; p<0.0332, **; p<0.0021, and ***; p<0.0002.

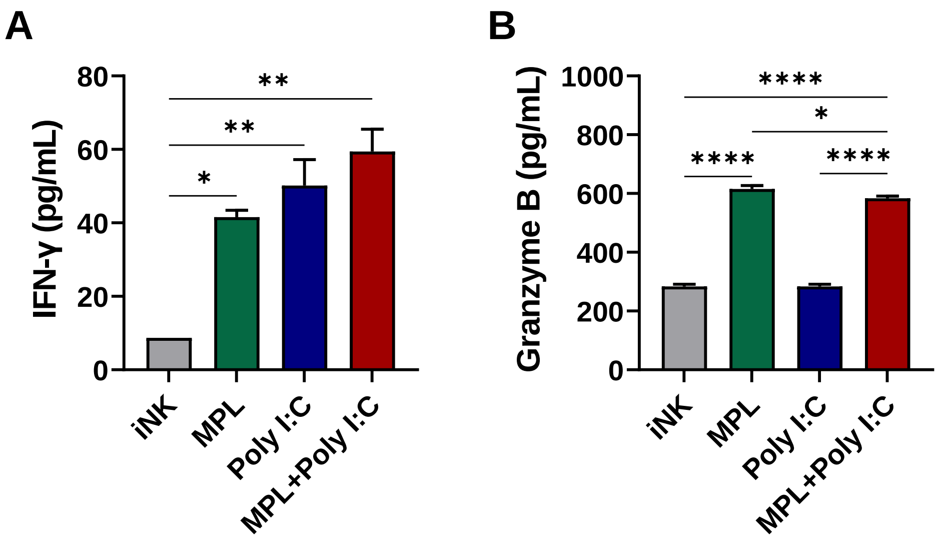

Supplementary Figure S5. Cytokine production by isolated NK cells after *in vitro* adjuvant treatment. NK cells were isolated from naïve spleen cells and treated with MPL (100 ng/ml), Poly I:C (1000 ng/ml), or MPL+PolyI:C (100 ng/ml + 1000 ng/ml) for 2 days. The levels of IFN-γ and granzyme B in the culture supernatants were measured by ELISA. The data were shown in mean ± SD. Statistical comparisons between groups were performed in One-way ANOVA and Tukey's multiple comparison test. *; 0.0332, **; 0.0021, and ****; 0.0001.

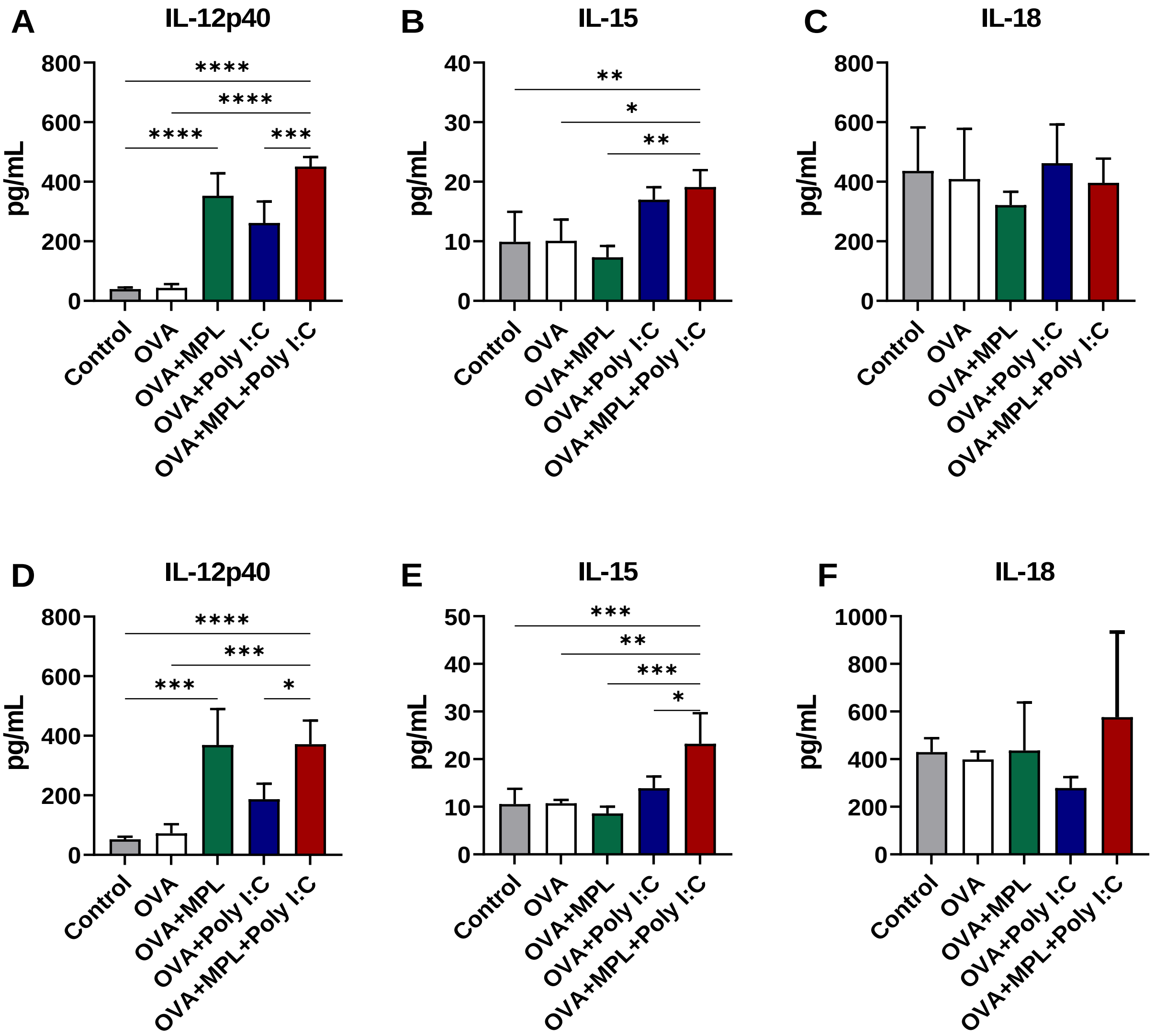

Supplementary Figure S6. Induction of cytokine production after stimulation by MPL and Poly I:C in the lungs of C57BL/6 mice at prime and boost dose. Lung homogenates were collected at day 1 post prime (**A-C**) and boost (**D-F**) immunizations and the levels of IL-12p40, IL-15 and IL-18 were measured by ELISA. The data were show in mean ± SD (n=5). Statistical comparisons between groups were performed in One-way ANOVA and Tukey's multiple comparison test. *; 0.0332, **; 0.0021, ***; 0.0002 and ****; 0.0001.
